# Comparison of Female Verzaschese and Camosciata delle Alpi Goats’ Hematological Parameters in The Context of Adaptation to Local Environmental Conditions in Semi-Extensive Systems in Italy

**DOI:** 10.3390/ani12131703

**Published:** 2022-06-30

**Authors:** Stella Agradi, Laura Menchetti, Giulio Curone, Massimo Faustini, Daniele Vigo, Luca Villa, Sergio Aurelio Zanzani, Rezart Postoli, Tana Shtylla Kika, Federica Riva, Susanna Draghi, Sebastiano Luridiana, Ivonne Archetti, Gabriele Brecchia, Maria Teresa Manfredi, Alessia Libera Gazzonis

**Affiliations:** 1 Department of Veterinary Medicine and Animal Sciences (DIVAS), University of Milan, Via dell’Università 6, 26900 Lodi, Italy; stella.agradi@unimi.it (S.A.); giulio.curone@unimi.it (G.C.); massimo.faustini@unimi.it (M.F.); daniele.vigo@unimi.it (D.V.); luca.villa@unimi.it (L.V.); sergio.zanzani@unimi.it (S.A.Z.); federica.riva@unimi.it (F.R.); susanna.draghi@unimi.it (S.D.); gabriele.brecchia@unimi.it (G.B.); mariateresa.manfredi@unimi.it (M.T.M.); 2 Department of Agricultural and Agri-Food Sciences and Technologies, University of Bologna, Viale Fanin 46, 40138 Bologna, Italy; laura.menchetti7@gmail.com; 3 Faculty of Veterinary Medicine, Agricultural University of Albania, Rr Paisi Vodica, Koder, 1029 Kamez, Albania; rezart.postoli@ubt.edu.al (R.P.); tana.shtylla@ubt.edu.al (T.S.K.); 4 Department of Veterinary Medicine of Sassari, University of Sassari, Via Vienna 2, 07100 Sassari, Italy; sluridiana@uniss.it; 5 Istituto Zooprofilattico Sperimentale della Lombardia e dell’Emilia Romagna, Via A. Bianchi, 9, 25124 Brescia, Italy; ivonne.archetti@izsler.it

**Keywords:** Verzasca goat, Alpine goat, reference intervals, melatonin, biodiversity, inter-breed genetic variability

## Abstract

**Simple Summary:**

Livestock biodiversity is being lost at an alarming rate. It is mainly due to genetic selection for desirable traits and the standardization of productions, but this has determined a dramatic reduction in intra- and inter-breed genetic variability. Autochthonous breeds represent a pivotal genetic resource thanks to their environment-specific adaptive features, especially related to rusticity, frugality, fertility, and longevity. In this context, it is of fundamental importance to deepen the knowledge about these breeds’ physiology and to take care of their health in the optic of conservation strategies. This study established hematological reference values in female goats of a cosmopolitan (i.e., Camosciata delle Alpi) and an Italian autochthonous (i.e., Verzaschese) breed reared in the same conditions. The influence of breed, age, and season on the hematological parameters was also investigated. The results have shown that variation due to breed, age, and season in blood parameters could be attributed both to physiological changes, such as the ones given by ageing and melatonin effect, and to adaptive genetic processes, for example, towards gastrointestinal parasitism. These findings can be helpful to provide evidence of the importance of recovering endangered/rare local breeds, which are a fundamental heritage for agricultural biodiversity, and local culture.

**Abstract:**

Local livestock breeds are pivotal to ensure sustainable agriculture and represent a real genetic resource in the context of genetic variability reduction. This study aimed at broadening the knowledge about hematological values in female Verzaschese and Camosciata delle Alpi goats (an Italian local and a cosmopolitan goat breed, respectively) and investigating factors affecting them (i.e., breed, age, season). Blood samples were collected monthly from 34 Verzaschese and 37 Camosciata delle Alpi female goats kept under the same semi-extensive farming system for a whole year. The main hematological parameters were evaluated, and descriptive as well as inferential statistical analyses were performed. Reference intervals for complete blood cell count were established and several inter-breed differences were found. In particular, most of the red blood cell parameters were higher in Verzaschese than Camosciata delle Alpi (*p* < 0.05) suggesting a greater gastrointestinal parasites’ resilience of the local breed. The age effect (*p* < 0.05) was consistent with the literature while the season effect (*p* < 0.05) could be explained by the melatonin immunostimulant action and gastrointestinal parasitism influence. Overall, differences in blood values could be attributed to physiological changes and adaptive strategies developed through centuries highlighting the remarkable rusticity and adaptation to the environment and farming system of the local breed.

## 1. Introduction

The theme of livestock biodiversity in recent years is becoming increasingly important in the context of agriculture, rural development, and food and nutrition security [1]. According to FAO’s “The State of the World’s Biodiversity for Food and Agriculture” report, biodiversity is the variety of life at genetic, species, and ecosystem levels [2]. To date, livestock biodiversity is being lost at an alarming rate [3]. For instance, according to this report, out of a total of 618 different goat breeds, just 306 were classified as not at risk, while the others were considered as at risk, with unknown risk level or extinct [4]. The high-yielding breeds are the most farmed at the world level and genetic selection has favored some desirable traits and the standardization of productions. However, this has caused a loss of intra- and inter-breed genetic variability [5] as well as a dramatic reduction in the number and consistency of local breeds. Local or autochthonous breeds are characterized by important features resulting from the close interaction of their genetic background and the environmental conditions where they have been living for centuries [1]. For example, in the DAD-IS (Domestic Animal Diversity Information System) 62 goat breeds adapted to mountainous terrain are recorded, 30 that are heat-tolerant, 7 humidity-tolerant, 14 cold-tolerant, and 20 adapted to water scarcity [1]. Moreover, the preservation and rational use of local breeds play a crucial role in maintaining biodiversity and valorization of local products [6,7,8]. In Italy, the goat sector has a high level of biodiversity, with nearly 50 different local breeds [9]; the autochthonous breeds are farmed for their milk [10,11,12], meat quality and adaptive features, especially for the rusticity, frugality, fertility, and longevity [13,14]. These features make local goat breeds the first choice in marginal areas where other domesticated species struggle to graze (such as mountains, wood, and foothill grazing), helping the preservation of the habitat and landscape integrity, reducing fire and flood risks, and promoting seedling establishment by reducing biomass accumulation [15,16,17].

For these reasons, it is of pivotal importance to take care of these animals’ health in the optic of conservation strategies. Previous studies highlighted differences in the resistance/resilience against diseases and gastrointestinal parasites between cosmopolitan (e.g., Saanen and Camosciata delle Alpi) and local goat breeds [18,19,20]. Knowledge of hematological reference values of several goat breeds is still missing at a worldwide level. Different physiological adaptations of local breeds to specific environments can also be seen in the hematological parameters varying between one breed to another, as already described in many African [21,22] and Asian [23,24,25,26] breeds. In Italy, the hematological profile has been investigated without specifying any reference intervals in Nicastrese [27], Messinese [28], Girgentana, and Aspromontana [29] breeds. Hematological reference values have been established only for a few Mediterranean goat breeds in one study (Aspromontana, Girgentana, Messinese, Maltese, and Argentata dell’Etna goats [30]). Moreover, it is fundamental to take into consideration the factors which could influence the hematological profile of a patient. In particular, it is now well known that the hematological parameters in goats undergo changes concerning many factors such as breed [23,29,30], age [22,31], sex [23,32], physiological/reproductive status [33,34,35], season [36,37,38], environmental and management factors [39,40,41], nutrition [42], and diseases [43,44,45,46]. Based on these differences, it is necessary to establish for every breed appropriate physiological reference intervals that could be used to evaluate the physiological or pathological status of the animal.

**Figure 1:**
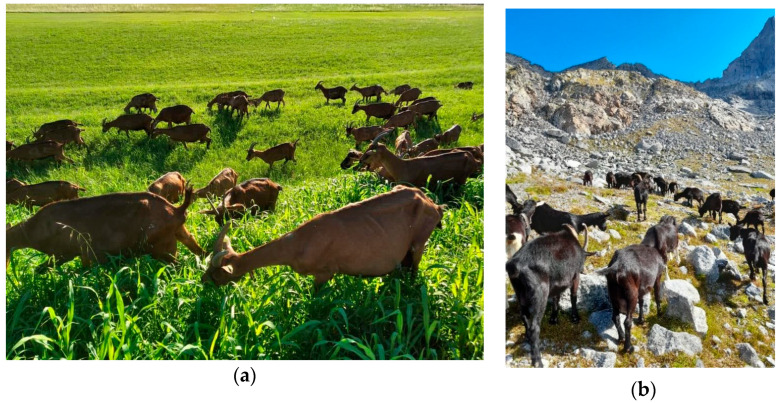
Camosciata delle Alpi (**a**) and Verzaschese (**b**) goats during free grazing (by courtesy of Mrs. Paola Rossi and Mr. Franco Del Bondio, respectively).

Camosciata delle Alpi or Alpine goat (Figure 1a) is a dairy breed native to the mountainous cantons of Bern, Freiburg, Glarus, and Graubünden in Switzerland. However, it is farmed in many European (especially in France, Italy, and Germany) and non-European countries. In Italy, it is mainly reared in the Alpine regions, especially in Piedmont and Lombardy. The Alpine goat is medium-large size, originally well adapted to the climate and mountainous geographical conditions. It has undergone a strong selection for quantitative milk production, neglecting other characteristics such as rusticity and resistance/resilience to diseases. The Verzaschese, also known as the Nera di Verzasca or Verzasca goat (Figure 1b), is a double aptitude native breed of the Verzasca Valley in the canton of Ticino in southern Switzerland. It is raised in that area and in some provinces of Lombardy and Piedmont, in the North of Italy. It is a medium-large size goat and is characterized by high rusticity and resistance both to high and low temperatures. In Italy, the Verzaschese is one of the 46 autochthonous goat breeds of limited distribution for which the national association of pastoralism (AssoNaPa) keeps a herd book.

To date, for both of these breeds, there is a lack of specific hematological reference values, and also the investigation of their complete blood cell count (CBC) in relation to age and season has never been reported in scientific literature. Just one study examined the variation in the hematological parameters through lactation in Camosciata delle Alpi goats [47], but reference intervals were not established and the sample size, as well as observations, were limited. We hypothesized that the baseline hematological parameters of the cosmopolitan (i.e., Camosciata delle Alpi) and local breed (i.e., Verzaschese) goats could differ, probably reflecting their different adaptative capacities to the specific Western alpine mountainous environment and the farming system. We also hypothesized that these parameters could be influenced by age and season.

Thus, this study aimed at establishing hematological reference values in female subjects of two breeds (one cosmopolitan and the other local): the Camosciata delle Alpi and the Verzaschese goat, reared in the same conditions. A second part of the study investigated the influence of breed, age, and season on the hematological parameters.

## 2. Materials and Methods

### 2.1. Farm Conditions and Animal Selection

A total of 71 female goats of Camosciata delle Alpi (*n* = 37) and Verzaschese (*n* = 34) breeds were enrolled for this study (Figure 1). 

All the animals were part of a flock reared in a unique structure under a semi-extensive farming system. Thus, the management conditions were the same for the two breeds. The farm was located at 980 m.s.l. (meters above mean sea level) in the mountains near Verbano Lake, in Varese province, Northern Italy. The area is characterized by cold winters when the temperature often reaches 0 °C, and dry and quite hot summers (the temperature reaches 25 °C). Therefore, the farming system had a prevalence of indoor housing in winter and free grazing on alpine pastures from spring to autumn, with indoor housing only during the night (or day in the hottest months). Thus, goats were exposed to natural photoperiod. During winter, the diet was based on *ad libitum* hay with increasing supplementation of concentrate from the dry period to early lactation (from 300 to 600 g/day). The birth season of the studied goats occurred from the 11th of January to the 21st of March. After kidding, goats were milked twice a day until September. From March to November, the goat’s diet was based on fresh forages on pastures (about 200 hectares) between 900 and 1550 m a.s.l. The main vegetal species eaten by goats in this context have been already described [48]. Anthelmintic treatment with netobimin 15 mg/kg was administered to all goats in November of the year before the study.

A clinical examination was performed on every goat before being included in the trial, and only apparently healthy subjects were submitted for blood collection (Camosciata delle Alpi, *n* = 37; Verzaschese, *n* = 34). The mean Body Condition Score (BCS) was 2.8/5 in Camosciata delle Alpi and 2.7/5 in the Verzaschese breed. Mean and min–max age for Camosciata delle Alpi goats were 5 (2–8) years, while for Verzaschese goats were 5 (3–12) years. Only pregnant nulliparous, primiparous, and pluriparous goats were included in the study. The mean rectal temperature varied in the range 38.5–39.7 °C. Goats that for any reason at the end of the experimental trial had less than 11 blood samples, were excluded from the study (Camosciata delle Alpi, *n* = 3; Verzaschese, *n* = 2). On the same day of blood collection, milk samples from each goat were collected and analyzed for basic lactation variables (milk yield, fat percentage, protein percentage, lactose percentage, and somatic cell count). The data derived from the milk analysis are reported and discussed in previous work by Agradi et al. [49]. Moreover, data about the parasitic load of the same goats included in our study and during the same period analyzed, are discussed in another study by Zanzani et al. [50].

### 2.2. Collection and Analysis of Blood Samples

Blood samples were collected monthly from every goat included in the study (Camosciata delle Alpi, *n* = 37; Verzaschese, *n* = 34) in the morning before feeding on the same day of the month from January to December by jugular venipuncture using 18G disposable needles into 3 mL sterile vacuum tubes containing K3EDTA as anticoagulant. Blood samples were refrigerated at 4 °C until analysis, which was performed within 8 hours from collection. On whole blood, the following parameters were determined: red blood cells (RBC), hemoglobin (HGB), packed cell volume (PCV), mean corpuscular volume (MCV), mean corpuscular hemoglobin (MCH), mean corpuscular hemoglobin concentration (MCHC), red cell distribution width (RDW), leucocyte count (WBC), neutrophil count (NEU), lymphocyte count (LYMPH), monocyte count (MONO), eosinophil count (EOS), basophil count (BAS), neutrophil percentage (NEU fraction), lymphocyte percentage (LYMPH fraction), monocyte percentage (MONO fraction), eosinophil percentage (EOS fraction), and basophil percentage (BAS fraction). N/L (neutrophils to lymphocytes) ratio was calculated as the ratio between neutrophil (NEU) and lymphocyte count (LYMPH).

Hematological analyses were performed in triplicate with the automated apparatus Cell-Dyn3500 (Abbott Laboratories, Abbott Park, IL, USA); veterinary software was implemented in the hematological apparatus.

### 2.3. Data Analysis

To determine the reference intervals, the “Guidelines for the determination of reference intervals in veterinary species” issued by the Quality Assurance and Laboratory Standards committee were followed, as reported by Friedrichs et al. [51]. First, extreme outliers (first quartiles − 3.0 interquartile range or third quartiles + 3.0 interquartile range) were identified and eliminated (Table S1). Then, the average for each subject and the mean intra-individual coefficient of variation (CV) were calculated for each parameter. The normality of the data distribution of each variable was assessed by a Kolmogorov–Smirnov test (Table S1) with Lilliefors significance correction and histograms. Finally, although most of the parameters showed a normal distribution, a robust method was chosen based on the 5th and 95th sample quantiles to determine reference limits and bootstrap to calculate 90% CI of reference limits. Moreover, mean, median (Mdn), first (Q1) and third (Q3) quartiles, and inter-individual CV were reported. 

The data were then analyzed by Mixed Linear Models with diagonal variance structure including month and goat as a repeated measure and as a random factor, respectively. These models evaluated the main effect of breed (2 levels: Camosciata delle Alpi and Verzaschese), season (4 levels: winter, spring, summer, and autumn), and age (as a continuous variable). Sidak corrections were used to obtain pairwise comparisons. The parameter estimates (b) with their standard error were also reported for the age effect. Parity and days in milk were not included in the models because of collinearity with age and season, respectively. Indeed, parity shows an effect on the hematological parameters similar to the one of age as, usually, goats have one birth per year starting from 1 year of age.

Statistical analyses were performed with SPSS Statistics version 25 (IBM, SPSS Inc., Chicago, IL, USA). Statistical significance occurred when *p* < 0.05.

## 3. Results

### 3.1. Hematological Parameters’ Reference Limits Related to Breed

The descriptive statistics, reference limits, and biological variation for the hematological parameters are presented in Table 1 and Table 2 for Camosciata delle Alpi and Verzaschese goat breeds, respectively. A higher biological variability, mainly intra-individual, can be noticed in the Camosciata delle Alpi than in Verzaschese goats.

### 3.2. Effects of Breed, Season, and Age on Hematological Variables

**Figure 2:**
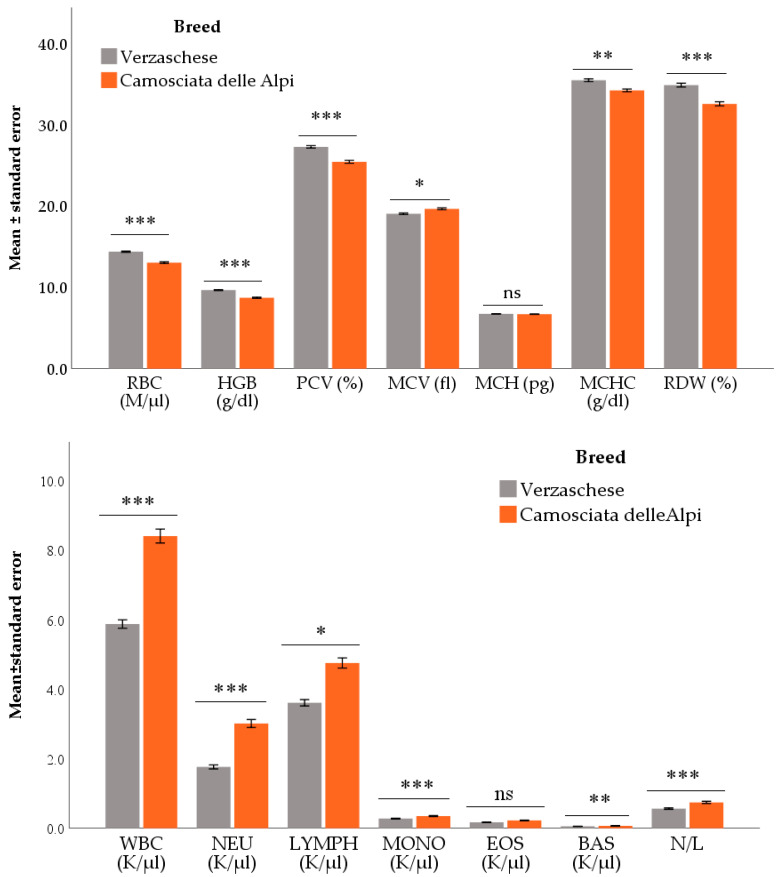
Main effect of breed on the evaluated parameters. Values are means and standard errors. *** *p* < 0.001, ** *p* < 0.01, * *p* < 0.05 Verzaschese vs. Camosciata delle Alpi. Ns = not significant (*p* < 0.05). Models also included Season and Age (as covariate). RBC = red blood cells; HGB = hemoglobin; PCV = packed cell volume; MCV = mean corpuscular volume; MCH = mean corpuscular hemoglobin; MCHC = mean corpuscular hemoglobin concentration; RDW = red cell distribution width; WBC = leucocyte count; NEU = neutrophil count; LYMPH = lymphocyte count; MONO = monocyte count; EOS = eosinophil count; BAS = basophil count; N/L = neutrophils to lymphocytes ratio.

The difference between Verzaschese and Camosciata delle Alpi goat breeds was statistically significant for all the red blood cells parameters (except MCH). Except for the MCV, they were higher in Verzaschese than Camosciata goats. As regards the white blood cells, WBC, NEU (both as count and fraction), LYMPH, MONO, and BAS counts, as well as N/L were higher in Camosciata delle Alpi than Verzaschese. Conversely, LYMPH fraction was higher in Verzaschese than Camosciata delle Alpi (Figure 2 and Figure S1, and Table 3).

**Figure 3:**
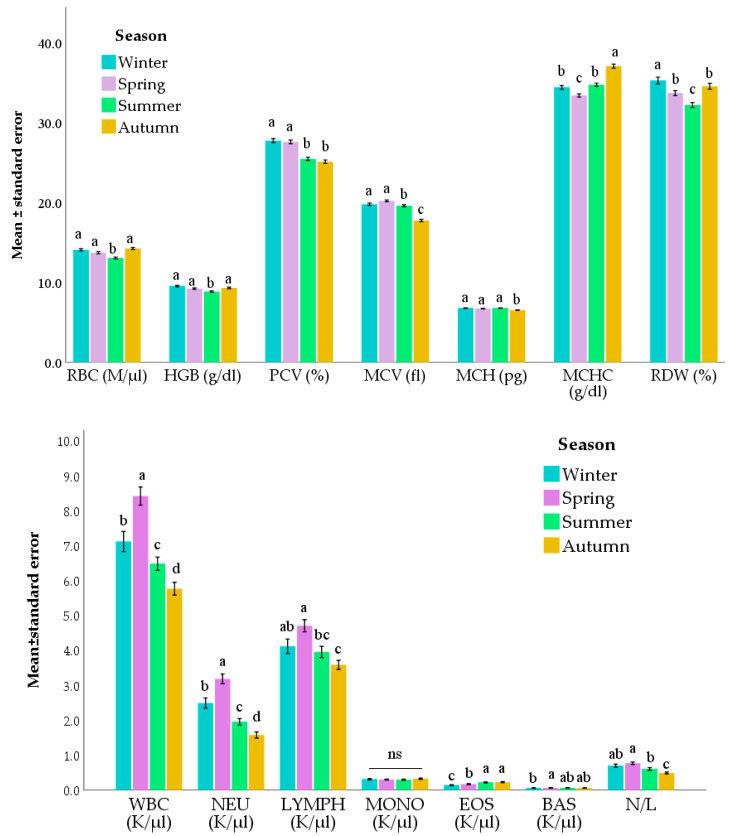
Main effect of season on the evaluated parameters. Values are means and standard errors. For each parameter, bars that do not share the same letter are significantly different (*p* < 0.05; multiple comparisons with Sidak correction). Models also included Breed and Age (as covariate). RBC = red blood cells; HGB = hemoglobin; PCV = packed cell volume; MCV = mean corpuscular volume; MCH = mean corpuscular hemoglobin; MCHC = mean corpuscular hemoglobin concentration; RDW = red cell distribution width; WBC = leucocyte count; NEU = neutrophil count; LYMPH = lymphocyte count; MONO = monocyte count; EOS = eosinophil count; BAS = basophil count; N/L = neutrophils to lymphocytes ratio.

Seasonal variations were also observed (Figure 3 and Figure S2, and Table 3). Most of the primary red blood cell variables (i.e., RBC, HGB, PCV, and RDW) were lower in summer than in winter and spring. MCV, MCH, and MCHC are exceptions as the lowest values were found in autumn or spring. In spring, the lowest values of LYMPH, MONO, EOS, and BAS fractions were also found. Conversely, spring showed the highest values of WBC, NEU (both as count and fraction), and N/L ratio. LYMPH count was also high in winter while EOS count showed a contrary trend (*p* <0.05), with the highest values in summer and autumn.

The association between age and hematological parameters was defined using the sign of b parameters (Table 3). A positive and significant effect was found for MCV, NEU, and BAS fractions and N/L, while a negative one for RBC, HGB, MCHC, RDW, WBC, and LYMPH (as count and fraction).

## 4. Discussion

### 4.1. Hematological Parameters’ Reference Limits Related to Breed

This is the first study that has investigated and compared the hematological values in Verzaschese and Camosciata delle Alpi goat breeds. The reference intervals obtained are in general narrower compared to the generic values reported by Schalm et al. [52], which do not refer to specific breeds but are usually consulted by the practitioners [53,54,55,56]. That testifies to the higher specificity given by reference limits built for specific breeds rather than for the entire species. In particular, the limits reported by Schalm et al. [52] for some of the red blood cells parameters (i.e., HGB, PCV, MCV, and MCH) resulted to be higher than what was found in Verzaschese and Camosciata delle Alpi goats. The establishment of hematological reference values for different breeds could be of considerable importance in the context of adaptation strategies and, in particular, for the *Capra hircus* which is adapted to many different climates and environmental conditions.

### 4.2. Comparative Hematological Changes Related to Breed, Age, and Season 

The examined breeds differed in several hematological parameters, mainly related to the red blood cells. In particular, Verzaschese showed higher mean values for RBC, HGB, PCV, MCHC, and RDW, while MCV was significantly lower than Camosciata delle Alpi goat. These differences could be partly due to subclinical anemia caused by the higher average gastrointestinal parasitic load of Camosciata delle Alpi goats than the Verzaschese goats, as reported by Zanzani et al. in a previous study which investigated the gastrointestinal nematode infections on the same goats and during the same period of our study [50]. Zanzani et al. evaluated the PCV trend along with the strongyle egg output. It was found that the higher strongyle egg output in Camosciata delle Alpi than Verzaschese goat corresponded to a lower PCV. That effect was ascribed to the presence of *H. contortus*, a hematophagus gastrointestinal parasite, whose presence was confirmed by coprocultures on goat feces [50]. Local goat breeds, such as Verzaschese, have shown at a worldwide level a greater resistance and resilience to diseases endemic to their area of origin than cosmopolitan breeds, especially concerning gastrointestinal parasites [18,57,58]. This can be explained by the fact that the semi-extensive farming system that characterizes the autochthonous breeds could result in a longer co-evolution between pathogen agent and host as well as in greater exposure to harmful organisms than cosmopolitan breeds [18,19,57,59].

Regarding the leucocyte parameters, N/L ratio is considered a good index of stress, with an increase in N/L ratio proportional to the level of glucocorticoid released [60,61]. Thus, the lowest N/L which was found in Verzasca goats suggests their greater adaptation to environmental stressors. Interestingly, an unexpected result was that both EOS count and EOS fraction had not shown any significant difference between breeds, notwithstanding the different average gastrointestinal parasitic load between the examined goats of the two breeds [50].

Age is another factor that could influence the value of some hematological parameters. As found in other breeds [30,35,52,62,63], RBC, HGB, and MCHC increased with the age. This physiological variation could be due to a greater oxygen-carrying capacity of the younger goats compared with the older ones and, as a consequence, a higher metabolic activity [64]. The trend of RDW values was in agreement with previous studies [56], showing a decline with age. Indeed, poikilocytosis is very common in goat species, especially in young subjects. Conversely, MCV increased with age. That could be a physiological variation to the decreasing trend of many of the erythrocytic parameters although it was in contrast with the study of Antunović et al. [47]. Regarding the leukocytic parameters, as shown previously in other goat breeds [22,30,63], WBC and LYMPH fractions decreased with age. Moreover, in agreement with the studies mentioned above [22,30,63], the NEU fraction increased with age and, as a consequence, N/L ratio had a positive trend. The positive trend of N/L ratio with age has already been demonstrated in human beings [65]. The physiological meaning for this aspect could be probably ascribed to the ability to cope with stressors which becomes dysregulated with aging.

Season too plays a pivotal role in influencing most of the hematological parameters. Several erythrocytic variables, such as RBC, HGB, PCV, and MCV showed lower values in summer (and autumn for PCV, MCV, and MCH). This decrease in the red blood cell parameters could be due to the higher gastrointestinal parasitism which is responsible for causing anemia. Indeed, mixed parasitic infections are common in goat species [56] and, among others, *H. contortus* was identified as responsible for causing a decrease in the PCV of the goats of this study [50]. Indeed, strongyle egg output in our sampled subjects was previously investigated by Zanzani et al. [50]. It resulted higher during summer and autumn than in other seasons. During these seasons, goats were left free to graze on pastures that were contaminated by eggs and larvae of gastrointestinal parasites due both to the overwintering ability of some parasitic species on grass and to the exit from parasite quiescence in the gastrointestinal tract of others [66]. That also explicates the EOS fraction pattern: the lowest values were reported in winter and spring, which are followed by an increase in summer and autumn. Eosinophils are immune cells that assist with the defense against parasites [67] and, consistently, they increased during the seasons when a higher strongyle egg output was demonstrated (i.e., summer and autumn [50]). 

Regarding the leukocytic parameters, WBC, NEU, and LYMPH counts had higher values in winter and spring than in summer and autumn. That could be the effect of the melatonin immunostimulating properties. Indeed, melatonin is a hormone with immunomodulatory activity, which has demonstrated positive effects on both innate and acquired immune functions in goats during short-daylength months [68]. Goats are short-day breeders and their reproductive activity is strictly linked to the relative duration of the preceding photoperiod, which biologically results in the melatonin release pattern [69]. Todini et al. [70] found that in two Italian goat breeds, at latitudes close to those of our study, the plasma melatonin nycthemeral profiles show higher values in winter and spring than in summer, and a significant difference among melatonin concentrations in months with similar day lengths but opposite photoperiods (April and August). In particular, in this investigation, higher concentrations of the hormone were found during spring than at the end of summer [70]. Thus, we could attribute the significant increment during winter and spring of WBC, NEU, and LYMPH counts to the physiological effect of photoperiod on goats. 

Finally, our results regarding the season effect could be used in the evaluation of the days in milk effect because of the collinearity among these two aspects. Considering this, our findings are in general consistent with what is already described in the literature [35,47]. In particular, in agreement with our results, red blood cell parameters showed a negative trend along lactation in the study conducted on Alpine goats by Antunović et al. [47]. Regarding white blood cell parameters, our study showed an increase in WBC, NEU, and LYMPH counts during the peri-partum period, and that is consistent with what was observed by Mbassa et al. [35].

This study has potential limitations especially concerning the “sample bias”. A larger number of subjects and farms and a longer interval of sampling time could improve the external validity of the results. It must be remembered that this would introduce another bias given by the farm management system. The statistical approach was nevertheless robust and followed the indications of the reference interval guidelines [51]. Moreover, including goats reared under the same management conditions may have reduced the influence on hematological variables of exogenous factors such as nutrition.

## 5. Conclusions

In conclusion, our findings confirmed that the hematological reference intervals of the local breed (Verzaschese) differ from that of the cosmopolitan one (Camosciata delle Alpi) and that these differences are indicative of greater resilience towards gastrointestinal parasitism and adaptation to environmental stressors of the local breed, probably as a result of adaptive strategies to the context of breeding developed through centuries. Hematological parameters changed also according to the animal’s age and season. In the latter case, an effect of melatonin could be speculated. The results here presented can add some knowledge to the definition of the health status of the two breeds. Moreover, these findings can be helpful to provide evidence of the importance of recovering endangered/rare local breeds, fundamental heritage for agricultural biodiversity, and local culture. Verzaschese goat represents an agricultural pillar for many regions in Northern Italy and Switzerland, and a breed with unique adaptive characteristics, like other livestock local breeds must be preserved, either by the optimal maintenance of subjects or the conservation of genetics.

## Figures and Tables

**Table 1:** Descriptive statistics, reference limits, and biological variation for hematological variables in Camosciata delle Alpi goats.

Parameter	Descriptive Statistics				Reference Limits (90% CI)		Biological Variation	
	Q1	Mdn	Q3	Mean	Lower	Upper	Intra-Individual CV (%)	Inter-Individual CV (%)
RBC (M/µL)	12.30	13.13	13.77	12.92	10.40 (9.87–1.41)	14.89 (14.28–5.04)	11	9
HGB (g/dL)	8.21	8.59	9.19	8.61	6.30 (5.96–7.74)	10.55 (9.52–10.84)	11	11
PCV (%)	24.10	24.96	26.62	25.15	20.50 (18.84–23.51)	28.67 (27.43–28.79)	10	8
MCV (fL)	18.77	19.50	20.61	19.67	17.11 (17.02–17.92)	21.87 (21.49–21.94)	7	7
MCH (pg)	6.53	6.64	6.90	6.68	6.30 (6.28–6.42)	7.14 (6.96–7.17)	6	3
MCHC (g/dL)	32.64	34.04	35.33	34.23	31.29 (31.02–31.96)	38.46 (37.22–38.75)	6	6
RDW (%)	30.15	33.06	34.87	32.65	27.10 (26.56–28.58)	36.85 (36.03–36.85)	10	9
WBC (K/µL)	7.48	8.81	9.84	8.66	4.45 (4.23–6.10)	11.97 (10.89–12.36)	29	23
NEU (K/µL)	2.25	3.18	3.84	3.12	1.62 (1.51–1.95)	5.11 (4.19–5.70)	51	32
LYMPH (K/µL)	3.28	4.11	6.13	4.62	2.12 (1.63–2.75)	7.86 (6.76–8.98)	31	37
MONO (K/µL)	0.27	0.37	0.46	0.38	0.21 (0.21–0.24)	0.70 (0.55-0.74)	54	35
EOS (K/µL)	0.15	0.18	0.29	0.22	0.09 (0.08–0.11)	0.45 (0.34-0.52)	74	46
BAS (K/µL)	0.05	0.07	0.08	0.07	0.02 (0.02–0.04)	0.12 (0.10–0.13)	64	37
NEU fraction (%)	28.38	36.56	44.02	36.91	18.65 (18.06–23.60)	59.83 (48.38–67.22)	36	30
LYMPH fraction (%)	45.88	54.86	61.00	53.47	29.58 (25.84–39.62)	75.74 (67.74–77.76)	24	22
MONO fraction (%)	3.77	4.94	6.11	5.06	2.29 (2.04–3.27)	8.54 (7.16–9.17)	63	33
EOS fraction (%)	1.74	2.69	3.36	2.91	1.08 (0.98–1.35)	6.96 (4.60–7.52)	77	53
BAS fraction (%)	0.60	0.75	1.08	0.86	0.36 (0.34–0.45)	1.56 (1.33–1.67)	66	41
N/L	0.51	0.77	1.03	0.81	0.27 (0.24–0.38)	1.63 (1.29–1.82)	56	47

Q1 = first quartile; Mdn = median; Q3 = third quartile; CV = coefficient of variation; RBC = red blood cells; HGB = hemoglobin; PCV = packed cell volume; MCV = mean corpuscular volume; MCH = mean corpuscular hemoglobin; MCHC = mean corpuscular hemoglobin concentration; RDW = red cell distribution width; WBC = leucocyte count; NEU = neutrophil count; LYMPH = lymphocyte count; MONO = monocyte count; EOS = eosinophil count; BAS = basophil count; NEU fraction = neutrophil percentage; LYMPH fraction = lymphocyte percentage; MONO fraction = monocyte percentage; EOS fraction = eosinophil percentage; BAS fraction = basophil percentage; N/L = neutrophils to lymphocytes ratio.

**Table 2:** Descriptive statistics, reference limits, and biological variation for hematological variables in Verzaschese goats.

Parameter	Descriptive Statistics				Reference Limits (90% CI)		Biological Variation	
	Q1	Mdn	Q3	Mean	Lower	Upper	Intra-Individual CV (%)	Inter-Individual CV (%)
RBC (M/µL)	14.07	14.42	14.93	14.30	11.99 (11.87–3.08)	15.78 (15.21–5.81)	7	7
HGB (g/dL)	9.31	9.67	10.14	9.61	7.80 (7.51–8.86)	11.03 (10.45–11.23)	7	8
PCV (%)	26.13	27.10	28.21	27.18	23.71 (22.93–25.29)	30.78 (29.69–30.95)	8	7
MCV (fL)	18.25	18.99	19.68	19.09	17.67 (17.57–17.92)	21.54 (20.52–21.75)	7	6
MCH (pg)	6.59	6.70	6.87	6.72	6.36 (6.35–6.46)	7.07 (6.98–7.09)	6	3
MCHC (g/dL)	34.64	35.43	36.91	35.47	32.46 (32.02–33.09)	38.38 (37.45–38.83)	7	5
RDW (%)	32.51	36.05	36.93	34.76	28.63 (28.47–30.22)	38.52 (37.43–38.82)	9	9
WBC (K/µL)	4.93	5.53	7.18	5.90	3.40 (3.19–4.25)	9.15 (7.89–9.54)	27	27
NEU (K/µL)	1.26	1.76	2.11	1.77	0.87 (0.87–1.02)	2.85 (2.45–3.19)	46	33
LYMPH (K/µL)	2.72	3.50	4.35	3.57	1.80 (1.79–2.20)	5.45 (5.12–5.58)	32	31
MONO (K/µL)	0.22	0.28	0.32	0.28	0.17 (0.16–0.18)	0.40 (0.37–0.41)	55	25
EOS (K/µL)	0.08	0.16	0.21	0.17	0.05 (0.05–0.06)	0.42 (0.26–0.51)	72	60
BAS (K/µL)	0.04	0.05	0.06	0.05	0.03 (0.02–0.03)	0.10 (0.08–0.10)	68	34
NEU fraction (%)	24.18	31.41	35.28	30.13	16.87 (16.73–20.04)	45.85 (37.78–49.30)	34	25
LYMPH fraction (%)	55.55	59.30	67.87	60.42	44.26 (43.21–49.15)	74.65 (71.86–74.81)	18	14
MONO fraction (%)	4.18	4.94	5.65	4.91	2.99 (2.85–3.43)	7.11 (6.13–7.59)	57	23
EOS fraction (%)	1.75	2.68	3.98	2.94	1.12 (1.02–1.35)	6.85 (4.38–9.51)	77	57
BAS fraction (%)	0.68	0.86	1.12	0.93	0.49 (0.49–0.58)	1.61 (1.33–1.70)	63	33
N/L	0.38	0.59	0.71	0.57	0.24 (0.23–0.31)	1.03 (0.85–1.24)	52	40

Q1 = first quartile; Mdn = median; Q3 = third quartile; CV = coefficient of variation; RBC = red blood cells; HGB = hemoglobin; PCV = packed cell volume; MCV = mean corpuscular volume; MCH = mean corpuscular hemoglobin; MCHC = mean corpuscular hemoglobin concentration; RDW = red cell distribution width; WBC = leucocyte count; NEU = neutrophil count; LYMPH = lymphocyte count; MONO = monocyte count; EOS = eosinophil count; BAS = basophil count; NEU fraction = neutrophil percentage; LYMPH fraction = lymphocyte percentage; MONO fraction = monocyte percentage; EOS fraction = eosinophil percentage; BAS fraction = basophil percentage; N/L = neutrophils to lymphocytes ratio.

**Table 3:** Results of the mixed linear model that analyzed the main effects of breed, season, and age on the evaluated parameters. For the age effect (included in the models as a continuous variable), the parameter estimates (b) with its standard error are also reported.

Variable	Breed Effect	Season Effect	Age Effect	
	*p*-Value	*p*-Value	b ± Standard Error	*p*-Value
RBC (M/µL)	<0.001	<0.001	−0.24 ± 0.05	<0.001
HGB (g/dL)	<0.001	<0.001	−0.17 ± 0.04	<0.001
PCV (%)	<0.001	<0.001	−0.23 ± 0.12	0.058
MCV (fL)	0.024	<0.001	0.20 ± 0.07	0.004
MCH (pg)	0.549	<0.001	−0.01 ± 0.01	0.878
MCHC (g/dL)	0.001	<0.001	−0.35 ± 0.09	<0.001
RDW (%)	<0.001	<0.001	−0.64 ± 0.14	<0.001
WBC (K/µL)	<0.001	<0.001	−0.35 ± 0.08	<0.001
NEU (K/µL)	<0.001	<0.001	0.04 ± 0.04	0.376
LYMPH (K/µL)	0.049	<0.001	−0.40 ± 0.07	<0.001
MONO (K/µL)	<0.001	0.170	−0.01 ± 0.01	0.411
EOS (K/µL)	0.096	<0.001	−0.01±0.01	0.107
BAS (K/µL)	0.001	0.008	−0.01 ± 0.01	0.969
NEU fraction (%)	<0.001	<0.001	2.13 ± 0.44	<0.001
LYMPH fraction (%)	<0.001	<0.001	−2.34 ± 0.50	<0.001
MONO fraction (%)	0.300	<0.001	0.13 ± 0.07	0.093
EOS fraction (%)	0.987	<0.001	0.02 ± 0.08	0.779
BAS fraction (%)	0.855	0.005	0.04 ± 0.17	0.014
N/L	<0.001	<0.001	0.06 ± 0.01	<0.001

RBC = red blood cells; HGB = hemoglobin; PCV = packed cell volume; MCV = mean corpuscular volume; MCH = mean corpuscular hemoglobin; MCHC = mean corpuscular hemoglobin concentration; RDW = red cell distribution width; WBC = leucocyte count; NEU = neutrophil count; LYMPH = lymphocyte count; MONO = monocyte count; EOS = eosinophil count; BAS = basophil count; NEU fraction = neutrophil percentage; LYMPH fraction = lymphocyte percentage; MONO fraction = monocyte percentage; EOS fraction = eosinophil percentage; BAS fraction = basophil percentage; N/L = neutrophils to lymphocytes ratio.

## Data Availability

The datasets in this study are available from the corresponding author on reasonable request.

## Supplementary Materials

The following supporting information can be downloaded at: https://www.mdpi.com/article/10.3390/ani12131703/s1, Figure S1: Main effect of breed on the white cells evaluated as fractions. Values are means and standard errors. *** *p* < 0.001 Verzaschese vs. Camosciata delle Alpi. ns = not significant (*p* < 0.05). Models also included Season and Age (as covariate). NEU = neutrophil per-centage; LYMPH = lymphocyte percentage; MONO = monocyte percentage; EOS = eosinophil percentage; BAS = basophil percentage; Figure S2: Main effect of season on the white cells evaluated as fractions. Values are means and standard errors. For each parameter, bars that do not share the same letter are significantly different (*p* < 0.05; multiple comparisons with Sidak correction). Models also included Breed and Age (as covariate). NEU = neutrophil percentage; LYMPH = lymphocyte percentage; MONO = monocyte percentage; EOS = eosinophil percentage; BAS = basophil percentage; Table S1: Significance of the Kolmogorov–Smirnov tests and numbers of outliers eliminated for each parameter.
