# Supplementary material for: Comparison of Female Verzaschese and Camosciata delle Alpi Goats’ Hematological Parameters in The Context of Adaptation to Local Environmental Conditions in Semi-Extensive Systems in Italy
Source: Animals (Basel). 2022 Jun 30;12(13):1703. doi: 10.3390/ani12131703 (PMC9264801; doi:10.3390/ani12131703)
Supplement: Supplementary file 1 [file animals-12-01703-s001.zip › animals-1725371-supplementary.pdf]

Supplementary materials

Table S1. Significance of the Kolmogorov-Smirnov Tests and numbers of outliers eliminated for each parameter.

Parameter	Sig. Kolmogorov-Smirnov*		Outliers (deleted)
	Camosciata delle Alpi	Verzaschese	
RBC (M/µl)	0.200	0.022	2
HGB (g/dl)	0.200	0.200	0
PCV (%)	0.200	0.200	1
MCV (fl)	0.200	0.200	0
MCH (pg)	0.200	0.200	4
MCHC (g/dl)	0.200	0.200	1
RDW (%)	0.200	0.001	1
WBC (K/µl)	0.200	0.176	0
NEU (K/µl)	0.200	0.200	7
LYMPH (K/µl)	0.100	0.200	1
MONO (K/µl)	0.200	0.200	21
EOS (K/µl)	0.002	0.200	10
BAS (K/µl)	0.200	0.036	7
NEU fraction (%)	0.200	0.200	0
LYMPH fraction (%)	0.200	0.200	0
MONO fraction (%)	0.200*	0.200	20
EOS fraction (%)	0.028	0.200	9
BAS fraction (%)	0.058	0.200	10
N/L	0.200	0.200	9

Null hypothesis: the set of data comes from a Normal distribution.
RBC = red blood cells; HGB = haemoglobin; PCV = packed cell volume; MCV = mean corpuscular volume; MCH = mean corpuscular haemoglobin; MCHC = mean corpuscular haemoglobin concentration; RDW = red cell distribution width; WBC = leucocyte count; NEU = neutrophil count; LYMPH = lymphocyte count; MONO = monocyte count; EOS = eosinophil count; BAS = basophil count; NEU fraction = neutrophil percentage; LYMPH fraction = lymphocyte per-centage; MONO fraction = monocyte percentage; EOS fraction = eosinophil percentage; BAS fraction = basophil percentage; N/L = neutrophils to lymphocytes ratio.

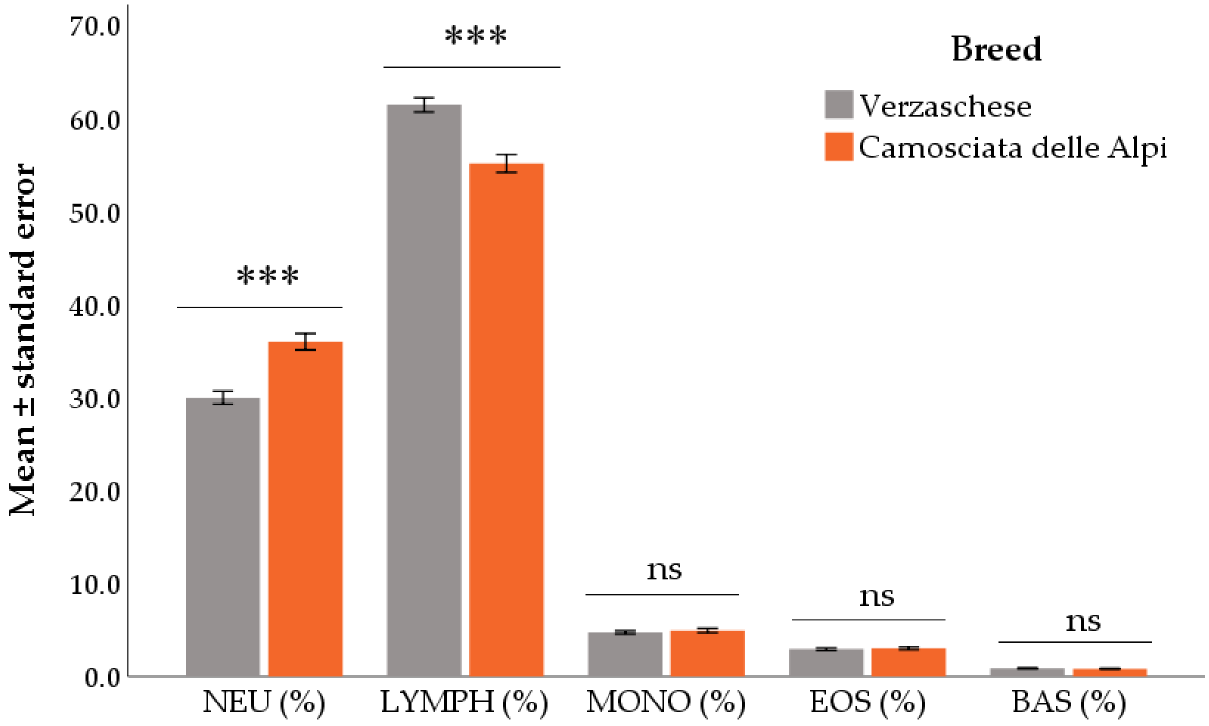

Figure S1. Main effect of breed on the white cells evaluated as fractions. Values are means and standard errors. ***p<0.001 Verzaschese vs Camosciata delle Alpi. ns = not significant (p < 0.05). Models also included Season and Age (as covariate). NEU = neutrophil percentage; LYMPH = lymphocyte percentage; MONO = monocyte percentage; EOS = eosinophil percentage; BAS = basophil percentage.

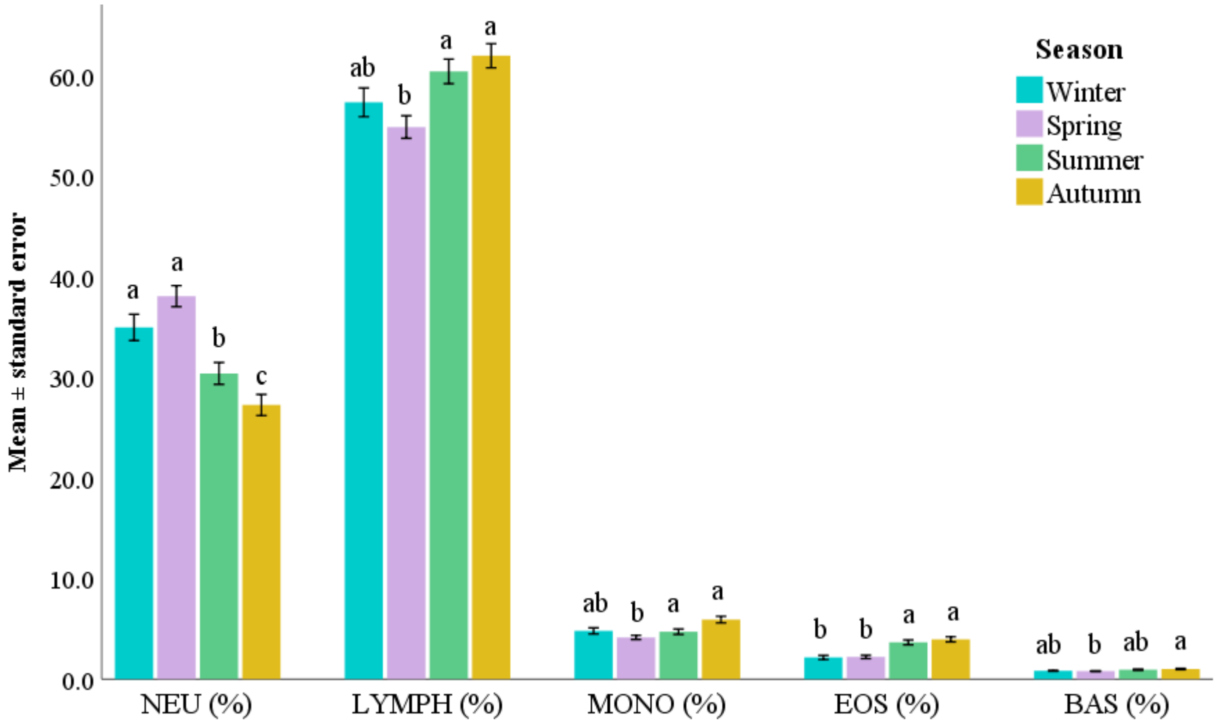

Figure S2. Main effect of season on the white cells evaluated as fractions. Values are means and standard errors. For each parameter, bars that do not share the same letter are significantly different ($p<0.05$; multiple comparisons with Sidak correction). Models also included Breed and Age (as covariate). NEU = neutrophil percentage; LYMPH = lymphocyte percentage; MONO = monocyte percentage; EOS = eosinophil percentage; BAS = basophil percentage.
